# Supplementary material for: Different sustained and induced alpha oscillations emerge in the human auditory cortex during sound processing
Source: Commun Biol. 2024 Nov 26;7:1570. doi: 10.1038/s42003-024-07297-w (PMC11599602; doi:10.1038/s42003-024-07297-w)
Supplement: Supplementary file 1 — Supplementary Information [file 42003_2024_7297_MOESM1_ESM.pdf]

SUPPLEMENTARY MATERIAL

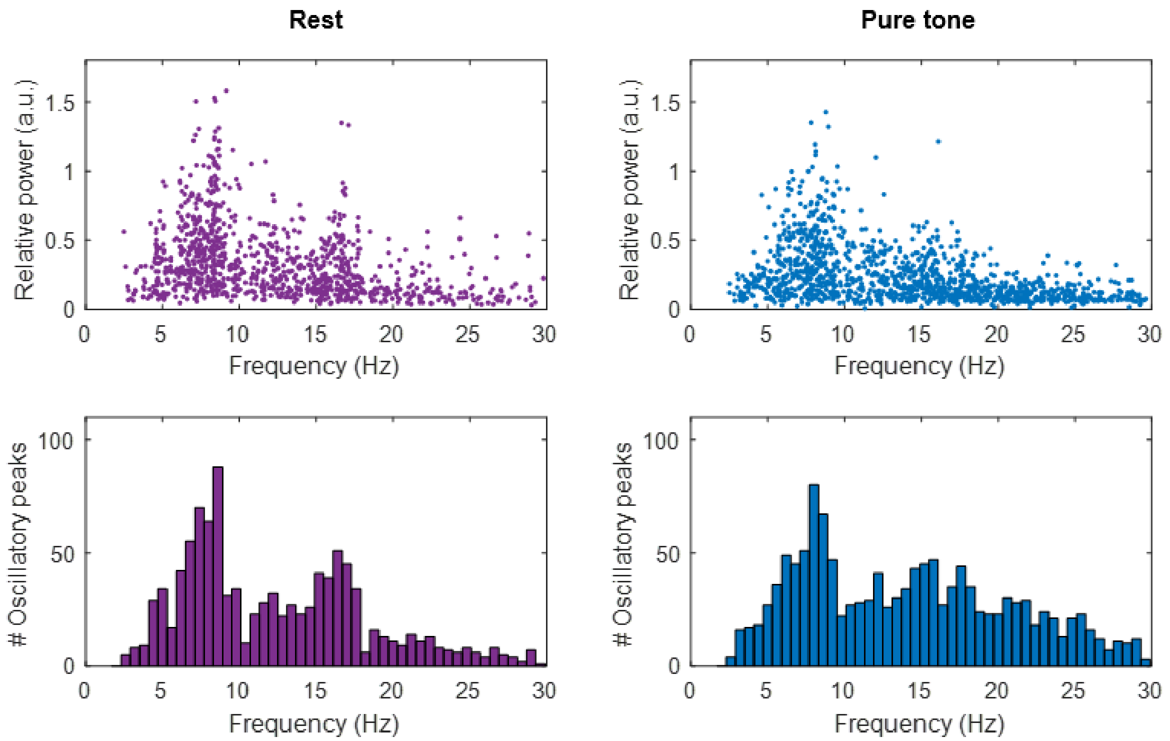

Supplementary Figure 1:

Distribution of peaks identified with the fooof approach for all the SEEG-ICs. There are two clear clusters of peaks, centered around 8 Hz and its first harmonic 16 Hz. The most prominent oscillations (i.e., peaks with higher relative power) were also centered between 5 and 10 Hz.

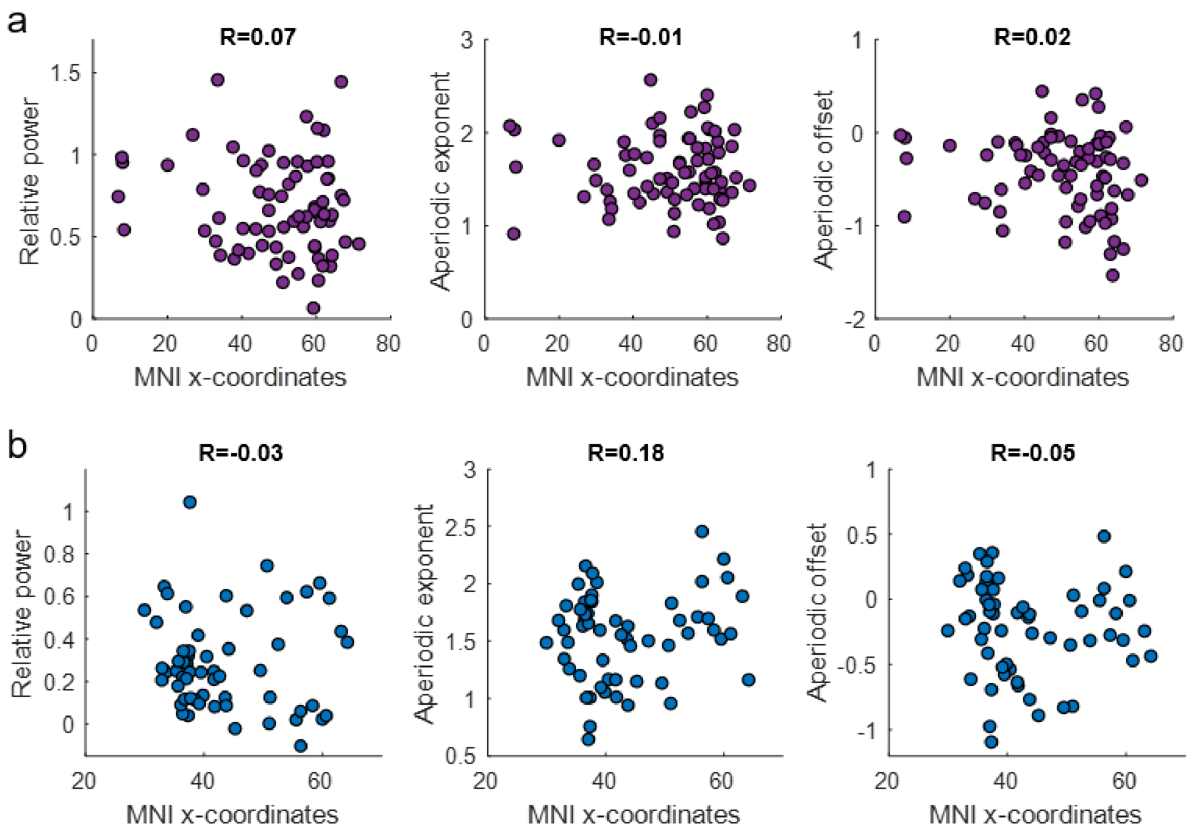

Supplementary Figure 2:

(a) Correlation between location of the oscillatory sources (position in the lateral-medial axis) and the main spectral features obtained from the fooof analysis: the relative power of the main oscillatory activity, the exponent and the offset of the aperiodic component. No significant correlations were found.

(b) Correlation between location of the evoked sources and the main spectral features.

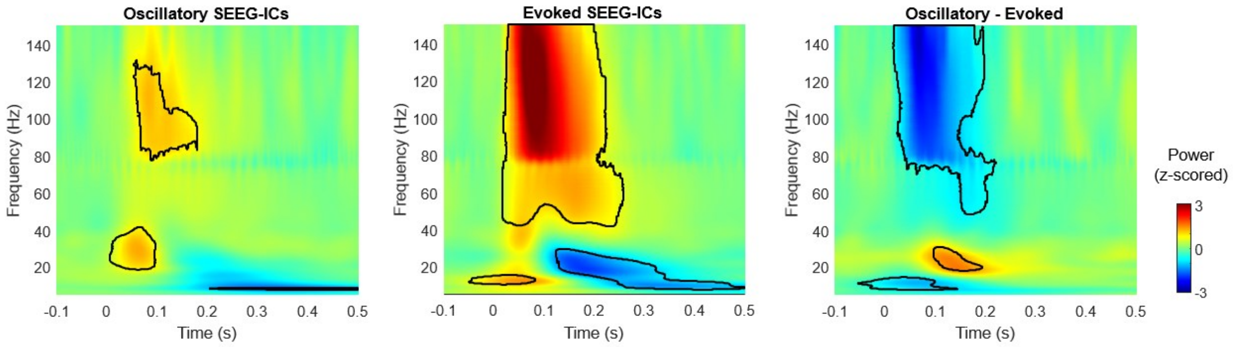

Supplementary Figure 3: Time-frequency response across patients during pure tone stimulation for oscillatory sources (left), evoked sources (middle) and difference between oscillatory and evoked sources. For patients with multiple oscillatory or evoked SEEG-ICs, we averaged them to have a single oscillatory and evoked source per subject. For left and middle panels, framed areas represent clusters of significant modulation of activity compared to baseline (-300 -200 ms, p<0.01, surrogate test, N=20). For right panel, delineated areas represent the clusters with significant difference (p<0.05 corrected with FDR, N=20).

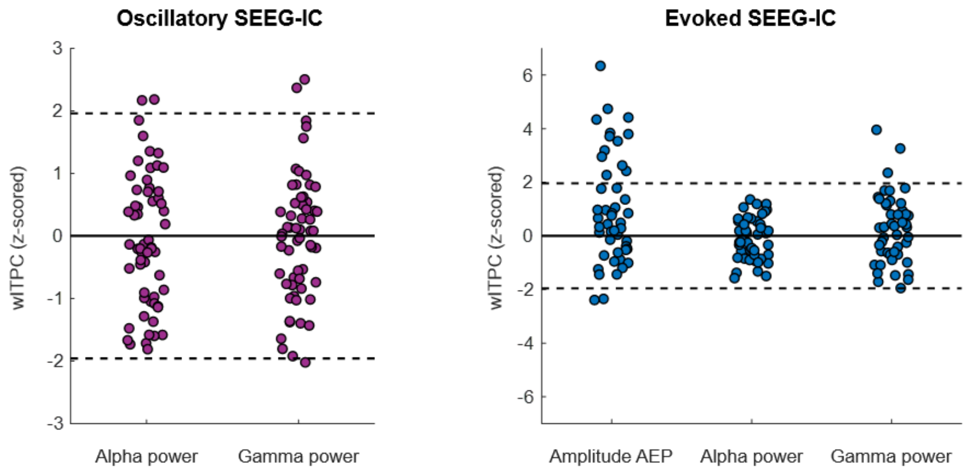

Supplementary Figure 4: Weighted ITPC between the instantaneous phase of alpha oscillations at stimulus arrival and three different features of the response: alpha (5-10 Hz) power, high-gamma (80-120 Hz) power and amplitude of the AEP. Each point represents one SEEG-IC. Dashed lines correspond to the significant threshold, stablished at 1.96. Values outside this rage were considered as significant.
