# Supplementary material for: Different sustained and induced alpha oscillations emerge in the human auditory cortex during sound processing
Source: Commun Biol. 2024 Nov 26;7:1570. doi: 10.1038/s42003-024-07297-w (PMC11599602; doi:10.1038/s42003-024-07297-w)
Supplement: Supplementary file 2 — Reporting Summary [file 42003_2024_7297_MOESM2_ESM.pdf]

nature portfolio

Corresponding author(s): DBPR COMMSBIO-24-3303

Last updated by author(s): Jun 26, 2024

Reporting Summary

Nature Portfolio wishes to improve the reproducibility of the work that we publish. This form provides structure for consistency and transparency in reporting. For further information on Nature Portfolio policies, see our Editorial Policies and the Editorial Policy Checklist.

Statistics

For all statistical analyses, confirm that the following items are present in the figure legend, table legend, main text, or Methods section.

n/a	Confirmed	
☐	☒	The exact sample size (*n*) for each experimental group/condition, given as a discrete number and unit of measurement
☐	☒	A statement on whether measurements were taken from distinct samples or whether the same sample was measured repeatedly
☐	☒	The statistical test(s) used AND whether they are one- or two-sided<br>*Only common tests should be described solely by name; describe more complex techniques in the Methods section.*
☐	☒	A description of all covariates tested
☐	☒	A description of any assumptions or corrections, such as tests of normality and adjustment for multiple comparisons
☐	☒	A full description of the statistical parameters including central tendency (e.g. means) or other basic estimates (e.g. regression coefficient) AND variation (e.g. standard deviation) or associated estimates of uncertainty (e.g. confidence intervals)
☐	☒	For null hypothesis testing, the test statistic (e.g. *F*, *t*, *r*) with confidence intervals, effect sizes, degrees of freedom and *P* value noted<br>*Give P values as exact values whenever suitable.*
☒	☐	For Bayesian analysis, information on the choice of priors and Markov chain Monte Carlo settings
☐	☒	For hierarchical and complex designs, identification of the appropriate level for tests and full reporting of outcomes
☐	☒	Estimates of effect sizes (e.g. Cohen's *d*, Pearson's *r*), indicating how they were calculated

Our web collection on statistics for biologists contains articles on many of the points above.

Software and code

Policy information about availability of computer code

Data collection	SEEG signal was recorded on a digital system (Natus Medical Incorporated)<br>Stimuli were presented using E-prime 1.1 (Psychology Software Tools Inc., Pittsburgh, PA, USA).
Data analysis	To determine the exact location of each electrode and contact, a co-registration of the postimplantation CT-scan with the preoperative MRI was performed for each patient using GARDEL (https://meg.univ-amu.fr/wiki/GARDEL:presentation).<br>All the analysis were performed on MATLAB v2022b (Mathworks, Natick, MA), using the FieldTrip toolbox (https://www.fieldtriptoolbox.org/)

For manuscripts utilizing custom algorithms or software that are central to the research but not yet described in published literature, software must be made available to editors and reviewers. We strongly encourage code deposition in a community repository (e.g. GitHub). See the Nature Portfolio guidelines for submitting code & software for further information.

Data

Policy information about availability of data

All manuscripts must include a data availability statement. This statement should provide the following information, where applicable:

- Accession codes, unique identifiers, or web links for publicly available datasets
- A description of any restrictions on data availability
- For clinical datasets or third party data, please ensure that the statement adheres to our policy

The conditions of our ethics approval do not permit public archiving of anonymized study data. Readers seeking access to the data should contact the lead author. Access will be granted to named individuals in accordance with ethical procedures governing the reuse of clinical data, including completion of a formal data sharing agreement.

Research involving human participants, their data, or biological material

Policy information about studies with human participants or human data. See also policy information about sex, gender (identity/presentation), and sexual orientation and race, ethnicity and racism.

Reporting on sex and gender	So far, no differences have been identified in the auditory cortex based on sex or gender. Therefore, we did not performed sex or gender-based analysis in this work.
Reporting on race, ethnicity, or other socially relevant groupings	No socially constructed or socially relevant categorization variables were used in this work
Population characteristics	See above
Recruitment	A total of 18 epileptic patients with an intracerebral EEG implantation were recruited. All the participants presented one electrode passing through the Heschl's gyrus and the Planum Temporale, targeting the thalamus. None of them had their epileptogenic zone within the auditory areas as identified by experienced epileptologists. Participants were selected based on this criterion and they do not belong to a consecutive series of experiments.
Ethics oversight	Institutional Review board of the French Institute of Health (IRB00003888)

Note that full information on the approval of the study protocol must also be provided in the manuscript.

Field-specific reporting

Please select the one below that is the best fit for your research. If you are not sure, read the appropriate sections before making your selection.

☐ Life sciences ☒ Behavioural & social sciences ☐ Ecological, evolutionary & environmental sciences

For a reference copy of the document with all sections, see nature.com/documents/nr-reporting-summary-flat.pdf

Behavioural & social sciences study design

All studies must disclose on these points even when the disclosure is negative.

Study description	We recorded electro-physiological activity (SEEG) from the auditory cortex of epileptic patients during 10 minutes of resting state and auditory stimulation (listening to pure tones). We then compared the brain dynamics between both conditions.
Research sample	A total of 18 patients (10 females) between 8 and 54 years old with pharmacoresistant epilepsy were recorded with SEEG during their period of presurgical evaluation at the Hôpital de laTimone (Marseille, France).
Sampling strategy	This work has been performed on epileptic patients implanted in the auditory cortex with intracerebral electrodes for presurgical evaluation. These recordings are unique are depend solely on clinical reasons. We included in this study all the patients that could be recorded during the duration of the project.
Data collection	Data was recorded using a digital system (Natus Medical Incorporated) at the Hôpital de laTimone (Marseille, France). A medical doctor and a researcher were present during the recordings. Participants were instructed either to rest or to passively listen to a set of pure tones.
Timing	All the data was collected between 2017 and 2020 during the presurgical evaluation period of the patients. All the recordings of each participant were performed the same day.
Data exclusions	No data were excluded

Non-participation	No participants dropped out/declined participation
Randomization	Participant were not allocated into several groups.

Reporting for specific materials, systems and methods

We require information from authors about some types of materials, experimental systems and methods used in many studies. Here, indicate whether each material, system or method listed is relevant to your study. If you are not sure if a list item applies to your research, read the appropriate section before selecting a response.

Materials & experimental systems

n/a	Involved in the study
☒	☐ Antibodies
☒	☐ Eukaryotic cell lines
☒	☐ Palaeontology and archaeology
☒	☐ Animals and other organisms
☒	☐ Clinical data
☒	☐ Dual use research of concern
☒	☐ Plants

Methods

n/a	Involved in the study
☒	☐ ChIP-seq
☒	☐ Flow cytometry
☒	☐ MRI-based neuroimaging

Plants

Seed stocks	*Report on the source of all seed stocks or other plant material used. If applicable, state the seed stock centre and catalogue number. If plant specimens were collected from the field, describe the collection location, date and sampling procedures.*
Novel plant genotypes	*Describe the methods by which all novel plant genotypes were produced. This includes those generated by transgenic approaches, gene editing, chemical/radiation-based mutagenesis and hybridization. For transgenic lines, describe the transformation method, the number of independent lines analyzed and the generation upon which experiments were performed. For gene-edited lines, describe the editor used, the endogenous sequence targeted for editing, the targeting guide RNA sequence (if applicable) and how the editor was applied.*
Authentication	*Describe any authentication procedures for each seed stock used or novel genotype generated. Describe any experiments used to assess the effect of a mutation and, where applicable, how potential secondary effects (e.g. second site T-DNA insertions, mosiacism, off-target gene editing) were examined.*
